# P247 and P523: Two *In Vivo*-Expressed Megalocytivirus Proteins That Induce Protective Immunity and Are Essential to Viral Infection

**DOI:** 10.1371/journal.pone.0121282

**Published:** 2015-03-27

**Authors:** Jian Zhang, Bao cun Zhang, Li Sun

**Affiliations:** 1 Key Laboratory of Experimental Marine Biology, Institute of Oceanology, Chinese Academy of Sciences, Qingdao, China; 2 University of Chinese Academy of Sciences, Beijing, China; National Cheng Kung University, TAIWAN

## Abstract

Megalocytivirus is a DNA virus with a broad host range among teleost fish. Although the complete genome sequences of a number of megalocytivirus isolates have been reported, the functions of most of the genes of this virus are unknown. In this study, we selected two megalocytivirus immunogens, P247 and P523, which were expressed during host infection and, when in the form of DNA vaccines (pCN247 and pCN523 respectively), elicited strong protectivity against lethal megalocytivirus challenge in a turbot (*Scophthalmus maximus*) model. Compared to control fish, fish vaccinated with pCN247 and pCN523 exhibited drastically reduced viral loads in tissues and high levels of survival rates. Immune response analysis showed that pCN247 and pCN523 (i) induced production of specific serum antibodies, (ii) caused generation of cytotoxic immune cells and specific memory immune cells that responded to secondary antigen stimulation, and (iii) upregulated the expression of genes involved in innate and adaptive immunity. To examine the potential role of P247 and P523 in viral infection, the expression of P247 and P523 was knocked down by siRNA. Subsequent *in vivo* infection study showed that P247 and P523 knockdown significantly impaired viral replication. Furthermore, whole-genome transcriptome analysis revealed that P247 and P523 knockdown altered the expression profiles of 26 and 41 viral genes, respectively, putatively participating in diverse aspects of viral infection. Taken together, these results indicate that P247 and P523 induce protective immunity in teleost and play fundamental roles essential to viral replication. These observations provide the first evidence that suggests a likely link between the protectivity of viral immunogens and their biological significance in viral replication.

## Introduction

Iridoviruses are a family of double-stranded DNA viruses ranging between 120–300 nm in diameter. The family has five genera named *Iridovirus*, *Chloriridovirus*, *Lymphocystivirus*, *Ranavirus*, and *Megalocytivirus* [1]. Of these genera, *Megalocytivirus* is relatively newly identified and ranked worldwide as an important pathogen to finfish [2,3]. In China, severe megalocytivirus infections have been reported in mandarinfish (*Siniperca chuatsi*) [4], large yellow croaker (*Pseudosciaena crocea*) [5], turbot (*Scophthalmus maximus*) [6], and rock bream (*Oplegnathus fasciatus*) [7]. In countries other than China, megalocytivirus-associated disease outbreaks have been documented in a large number of farmed fish including red sea bream (*Pagrus major*) [8–10], turbot [11], flounder (*Paralichthys olivaceus*) [12], sea perch (*Lateolabrax japonicas*) [13], and rock bream [14]. Sequence analysis of megalocytivirus isolates from different hosts around the world revealed that *Megalocytivirus* is separated distinctly from the other genera of the *Iridovirus* family, and that the members within the *Megalocytivirus* genus possess highly conserved genetic features such as genome size (approximately 110 kb), GC content (53–55%), and gene number (115 to 124) [15–20]. However, the functions of the genes of megalocytivirus remain essentially unknown.

DNA vaccine is a genetic vaccine based on the gene that encodes an antigenic protein of a pathogen. It is carried on a plasmid, which, upon administration into the target animal, enables the expression of the vaccine gene via the transcription and translation machinery of the host animal [21,22]. The expressed antigen then induces specific immune response that protects the animal against infection of the relevant pathogen. DNA vaccine has been studied widely for the control of various diseases associated with viral pathogens, including fish viral pathogens such as infectious pancreatic necrosis virus (IPNV), viral hemorrhagic septicemia virus (VHSV), and Koi herpes virus disease (KHVD) [23–28]. For the *Iridovirus* family, DNA vaccines have been documented against red sea bream iridovirus and rock bream iridovirus, both belonging to the *Megalocytivirus* genus [29,30].

In previous studies, we isolated the fish megalocytivirus RBIV-C1 and characterized its host range and genomic sequence [7,20]. RBIV-C1 is highly pathogenic to turbot and rock bream and contains a genome of 112 kb with 119 open reading frames (ORFs). The ORFs of RBIV-C1 share high levels of sequence identities with the ORFs of other megalocytivirus isolates, notably orange-spotted grouper iridovirus (OSGIV), rock bream iridovirus (RBIV), and turbot reddish body iridovirus (TRBIV). In the present study, we selected two ORFs of RBIV-C1 with strong immunoprotective property as DNA vaccines and examined the immune response induced by these vaccines in a turbot model. In addition, we also investigated the potential role of these two ORFs in viral replication. Turbot was used as an animal model in this study because it is a natural host of megalocytivirus and an important economic species farmed widely in China as well as many other countries. As such, the results of the study may be applied directly to the control of megalocytivirus infection in aquaculture.

## Materials and Methods

### Ethics statement

Live animal researches were performed in accordance with the "Regulations for the Administration of Affairs Concerning Experimental Animals" promulgated by Shandong Province. The study and the mortality aspects of the protocol were approved by the Ethics Committee of Institute of Oceanology, Chinese Academy of Sciences.

### Fish

Clinically healthy juvenile turbot (*Scophthalmus maximus*) (average 12.3 g) were purchased from a local fish farm (Haiyang, Qingdao, China). The fish were maintained at 20°C and fed with commercial dry pellets as described previously [30]. Before experiment, the fish were verified to be pathogen free as described previously [30]. ELISA detected no serum antibodies against common fish pathogens. Before collection of tissues, fish were euthanized with tricaine methanesulfonate (Sigma, St. Louis, USA), and the spinal cord of the fish was then severed with a scalpel.

### Construction of DNA vaccine plasmids

The primers used in this study are listed in Table 1. The DNA vaccine plasmids pCN247 and pCN523, which express His-tagged P247 and P523 respectively, were constructed as follows. ORF107 and ORF86 (GenBank accession numbers AGG37986 and AGG37965 respectively) of megalocytivirus RBIV-C1, which encode P247 and P523 respectively, were amplified by PCR with the primer pairs P247F1/P247R1 and P523F1/P523R1 (Table 1) respectively. The PCR products were inserted to pEASY-Simple-T (TransGen Biotech, Beijing, China), and the vaccine genes were retrieved from the recombinant plasmids by digestion with EcoRV/SmaI and inserted into pCN3 [31] at the SmaI site as described previously [30]. Endo-Free Plasmid Kit (Omega Bio-tek, Doraville, USA) was used to prepare Endotoxin-free plasmid DNA. The quality of the DNA was analyzed as reported previously [30].

**Table 1:** Primers used in this study.

Primers	Sequences(5’-3’) ^a^
CNF1	CTTGCGTTTCTGATAGGCACCTA
HisR	GTGGTGGTGGTGGTGGTG
P247F1	GATATCGCCACCATGTTATTCCACGCCGAGA (EcoRV)
P247R1	GCGCGATATCCACATGCACCTCTACGGTG (EcoRV)
P247F2	GATATCATGGTGCACGTGATCCACGAC (EcoRV)
P247R2	GATATCCATCTTAGCCAGCTTCAGGATG (EcoRV)
P247F3	GACAGTTCTATAACACCGCAGTATC
P247R3	GCACGCCCGTCTTAGTATCG
P523F1	GGGCCCGCCACCATGGAAGTGGACATTTGCT (SmaI)
P523R1	GCGGGCCCTTGCTGCATTTGCCTAGT (SmaI)
P523F2	GATATCATGTCGCGGTTTGTCACTGAG (EcoRV)
P523R2	GATATCTGGGTCCAGTGTGTCGTC (EcoRV)
P523F3	CAAGTACCAAGCACCATCAGAAC
P523R3	ATAAGGTTATCAAGCAGGCTGTTAC

^a^Underlined nucleotides are restriction sites of the enzymes indicated in the brackets at the ends.

### Indirect immuno-fluorescence (IFA) analysis to examine expression of vaccine genes in transfected cells

Grunt Fin (GF) cells {purchased from American Type Culture Collection (ATCC), USA} were cultured at 24°C in DMEM medium/high glucose (HyClone, Logan, USA) supplemented with 20% fetal bovine serum (FBS) and 1% penicillin-streptomycin according to the instruction of the ATCC supplier. For transfection, the cells were seeded into 24-well culture plates at a concentration of 10^5^ cells/well and grown at 24°C until 80% confluency. The cells were then washed three times with opti-MEM medium (Invitrogen, Carlsbad, USA) and overlaid with 1 ml opti-MEM medium. One microgram pCN247, pCN523, or pCN3 was mixed with 2 μl Lipofectamine LTX and 1 μl PLUS (Invitrogen, Carlsbad, USA) in 50 μl opti-MEM medium, and the mixture was added to the cells. After 4 h incubation at 24°C, the cells were washed as above and overlaid with fresh DMEM medium containing 10% FBS. The cells were incubated at 24°C for 48 h. After incubation, the cells were fixed with 70% ethanol for 30 min at 4°C and washed three times with PBS. The cells were incubated with mouse anti-His monoclonal antibody (Bioss, Beijing, China) (1/1000 dilution) for 2 h at 37°C and then with FITC-labeled goat anti-mouse IgG (Tiangen, Beijing, China) (1/1000 dilution) for 1 h at 37°C. The cells were washed as above and examined with a fluorescence microscope (Nikon E800, Japan).

### Preparation of recombinant proteins

To obtain recombinant P247 (rP247) and P523 (rP523), the plasmids pEt247, and pEt523, which express P247 and P523 respectively, were constructed as follows. The coding sequences of P247 (residues 47–217), and P523 (residues 115–305), were amplified by PCR with the primer pairs P247F2/P247R2 and P523F2/P523R2 respectively (Table 1). The PCR products were inserted to pEASY-Simple-T as above, and the fragments containing P247 and P523 were retrieved from the recombinant plasmids by digestion with EcoRV. The retieved P247 and P523 fragments were inserted into pET259 [32] as described previously [33], resulting in pEt247 and pEt523. Protein preparation was performed as described previously [33] using Ni-NTA agarose (QIAGEN, Valencia, USA). The purified proteins were dialyzed against PBS for overnight and analyzed by sodium dodecyl sulfate-polyacrylamide gel electrophoresis. The proteins were stained with Coomassie brilliant blue R-250 (S1 Fig.). The concentration of the proteins was determined using BCA Protein Assay Kit (Sangon Biotech, Shanghai, China).

### Antibody preparation

To prepare anti-rP247 and anti-rP523 antibodies, adult rats (purchased from the Institute for Drug Control, Qingdao, China) were immunized via subcutaneous injection with rP247 or rP523 mixed in complete Freund’s adjuvant. The rats were boosted at 14 and 28 days after the initial immunization. The rats were bled 7 days after the last boost, and sera were obtained from the blood. The titer and specificity of the serum antibodies were determined by enzyme-linked immunosorbent assay and Western immunoblot analysis as described previously [34].

### Detection of P247 and P523 expression in megalocytivirus-infected fish

Turbot were infected via intraperitoneal (i.p.) injection with megalocytivirus RBIV-C1 [7] (1 × 10^5^ copies/fish) or PBS. At 5 days post-infection, peripheral blood leukocytes (PBL) were collected from the fish as described previously [35]. The cells were fixed with 70% pre-cooled ethanol for 30 min at 4°C. The cells were washed three times with PBS, and anti-rP247 and anti-rP523 antibodies (1/1000 dilution) prepared above were added to the cells. The cells were then treated as described previously [36] by washing three times with PBS and adding with fluorescein isothiocyanate (FITC)-labeled goat anti-rat IgG (Bioss, Beijing, China) (1/1000 dilution). The cells were incubated, washed, and resuspended in PBS as above. The cells were subjected to microscopic examination with a fluorescence microscope (Nikon E800, Japan).

### Vaccination

Vaccination was performed as reported previously [30]. Each of the vaccine plasmids was resuspended in PBS to the concentration of 400 μg/ml. Four groups (N = 70) of turbot (described in the section “Fish”) were injected intramuscularly (i.m.) with 50 μl pCN247, pCN523, pCN3, or PBS. The fish were maintained normally as described above in the section of “Fish”. At 7 days post-vaccination, 5 fish were taken from each group and used for examination of plasmid presence/expression of vaccine genes in fish tissues. At one month post-vaccination, 10 fish from each group were used for serum antibody analysis, and the remaining fish were challenged via i.p. injection with 50 μl megalocytivirus RBIV-C1 that had been suspended in PBS to 2 × 10^6^ copies/ml. At 24 h post-challenge, 5 fish from each group were examined for immune gene expression. At 3 and 5 days post-challenge, 10 fish (5/time point) from each group were examined for viral loads in spleen as described previously [7]. The remaining 40 fish were monitored daily (7 AM to 10 PM) for mortality over a period of one month. Moribund fish with the clinical signs of hemorrhages at the mouth, abdomen, and fins were picked out and euthanized with an overdose of tricaine methanesulfonate. However, since not all fish died of infection exhibited apparent clinical signs and some fish with mild symptoms (such as hemorrhage at the abdomen or fins only) could eventually survive, humane endpoint procedures could not be performed on all fish counted as dead. As a result, approximately 32% of the total mortality occurred naturally without euthanasia, which included (i) the fish died without the apparent clinical signs defined above, and (ii) the fish that may have developed full clinical signs at the un-monitored nighttime and died before 7 AM of the next day. Dying fish were randomly selected for the examination of virus in the liver, kidney, and spleen as described above. Relative percent of survival (RPS) was calculated as reported previously [30]. The vaccination experiment was performed three times (preliminary trial included).

### Detection of vaccine plasmids and expression of vaccine genes in fish tissues

At 7 days post-vaccination, tissues were taken from vaccinated fish and used for DNA and RNA extraction as reported previously [30]. PCR detection of pCN247, pCN523, and pCN3 was performed with the primer pairs P247F1/HisR, P523F1/HisR, and CNF1/HisR respectively (Table 1). Expression of the vaccine genes from pCN247 and pCN523, was performed by RT-PCR as described previously [33] with the primer pairs P247F1/HisR and P523F1/HisR, respectively (Table 1). P247F1 and P523F1 are specific to P247 and P523 respectively, while HisR is specific to the His sequence in the plasmid.

### Quantitative real time reverse transcription-PCR (qRT-PCR)

At 24 h post-challenge, spleen tissue was removed from the vaccinated fish (5/group). qRT-PCR was performed in an Eppendorf Mastercycler (Eppendorf, Hamburg, Germany) with SYBR Premix Ex Taq Kit (Takara, Dalian, China) as described previously [30]. The internal refernce gene was RNA polynerase II subunit D (RPSD) [37]. The primers of qRT-PCR have been reported previously [38]. The assay was performed three times.

### Enzyme-linked immunosorbent assay (ELISA)

Sera were collected from fish (5 from each group) vaccinated with pCN247, pCN523, pCN3, and PBS at one month post-vaccination respectively. Sera were diluted serially in 2-fold in PBS containing 1% bovine serum albumin (BSA). ELISA assay was preformed as reported previously [30]. The assay was performed three times.

### Cytotoxicity of PBL

The assay was performed using Cytotoxicity Detection Kit {(lactate dehydrogenase (LDH) assay} (Roche Applied Science, Indianapolis, USA) according to the manufacturer’s instructions. Briefly, target PBL were prepared from turbot infected with megalocytivirus RBIV-C1 for 5 days, and effector PBL were prepared form turbot vaccinated with pCN247, pCN523, or pCN3 as described previously [35]. The cells were suspended in L-15 medium (Invitrogen, Carlsbad, USA) containing 10% FBS. The effector cells were mixed with the target cells at the ratio of 50:1 in a 96-well cell culture plate. After incubation at 24°C for 24 h, the plate was centrifuged, and the cell-free supernatant was collected. Aliquots (100 μl/well) of the supernatant were transferred to a fresh 96-well plate. To determine the LDH activity in the supernatant, an equal volume of freshly prepared reaction mixture (from the above LDH kit) was added to the plate. The plate was incubated in the dark at room temperature for 30 min, and absorbance at 492 nm was measured. Cytotoxicity was calculated using the following formula: cytotoxicity (%) = (exp. Value-low control)/(high control–low control) × 100%. The assay was performed three times.

### Proliferative activity of PBL

PBL from turbot vaccinated with pCN247, pCN523, or pCN3 were prepared as reported previously [35]. The cells were distributed into 96-well tissue culture plates (∼1 × 10^5^ cells/well) containing L-15 medium (Invitrogen, Carlsbad, USA) with 10% FBS and 1% penicillin and streptomycin. rP247, rP523, or ConA (Sigma-Aldrich, St. Louis, USA) was added to the cells at the final concentration of 40 μg/ml, 40 μg/ml, or 80 μg/ml. L-15 medium was added to the control cells. The cells were incubated at 24°C for 48 h and added with 20 μl of 5 mg/ml MTT {3-(4,5)-dimethylthiahiazo (-z-y1)-3,5-di-phenytetrazoliumromide} (Sangon, Shanghai, China). After incubation at 28°C for 4 h, 200 μl DMSO was added to the plate to dissolve the reduced formazan. The plate was then read at 490 nm with a microplate reader. Stimulation index was defined as fold increase in the proliferation of antigen-treated cells compared to that of the untreated control cells. The assay was performed three times.

### RNA interference (RNAi)

- **Selection of effective siRNA.** RNAi was performed with DNA vector-based siRNA technology. To select siRNA with interfering effect on the expression of P247, three different siRNA targeting P247 were inserted into the siRNA expression vector pRNAT-CMV3.1 (GenScript, Piscataway, USA) at BamHI/AlfII sites, resulting in plasmids psiP247–1, psiP247–2, and psiP247–3. Similarly, three different siRNA targeting P523 were inserted into pRNAT-CMV3.1, resulting in plasmids psiP523–1, psiP523–2, and psiP523–3. In addition, the plasmid psiCR, which expresses a scramble siRNA, was constructed in the same fashion. To examine the efficiency of these siRNA plasmids, eight groups of turbot (N = 5) were injected i.m. with each of the plasmids (20 μg/fish) or with PBS. At 2 days post-plasmid administration, the fish were infected via i.p. injection with megalocytivirus RBIV-C1 (10^6^ copies/fish). At 5 days post-infection, spleen was taken under aseptic conditions and examined for the transcription of P247 and P523 by qRT-PCR as described above with the primer pairs P247F3/P247R3 and P523F3/P523R3 respectively (Table 1). The plasmids with the strongest inhibitory effect on P247 and P523 expression were re-named psiP247 and psiP523 respectively. This screening experiment was performed three times. The siRNA sequences expressed by psiP247, psiP523, and psiCR are 5’- CGTTTGTAGCGTCTTGCAAA-3’, 5’- CGCACCACTAGATGCGGCCGA-3’, and 5’- CGACCGTCGCGTTAGCTGGTA3’ respectively.
- **Effect of siRNA on viral replication.** Turbot were administered with psiP247, psiP523, psiCR, and PBS and challenged with megalocytivirus RBIV-C1 as above. At 3 days and 5 days post-challenge, spleen was taken under aseptic conditions and examined for viral load by absolute quantitative real time PCR as reported previously [7]. The experiment was performed three times.
- **Effect of siRNA on viral gene expression.** Turbot were administered with psiP247, psiP523, psiCR, and PBS and challenged with megalocytivirus RBIV-C1 as above. At 5 days post-challenge, spleen was taken under aseptic condition and used for total RNA extraction as described above. The expression of the 119 genes of megalocytivirus RBIV-C1 was then determined by qRT-PCR as above with specific primers [20]. The experiment was performed three times.

### Statistical analysis

The experiments were performed three times. Statistical analyses were carried out with the SPSS 17.0 package (SPSS Inc., Chicago, IL, USA). Chi-square test with Yates’ correction was used for mortality analysis, and analysis of variance (ANOVA) was used for all other analyses. In all cases, the significance level was defined as *P* < 0.05.

## Results

### Selection of megalocytivirus genes encoding protective immunogens

**Fig 1:**
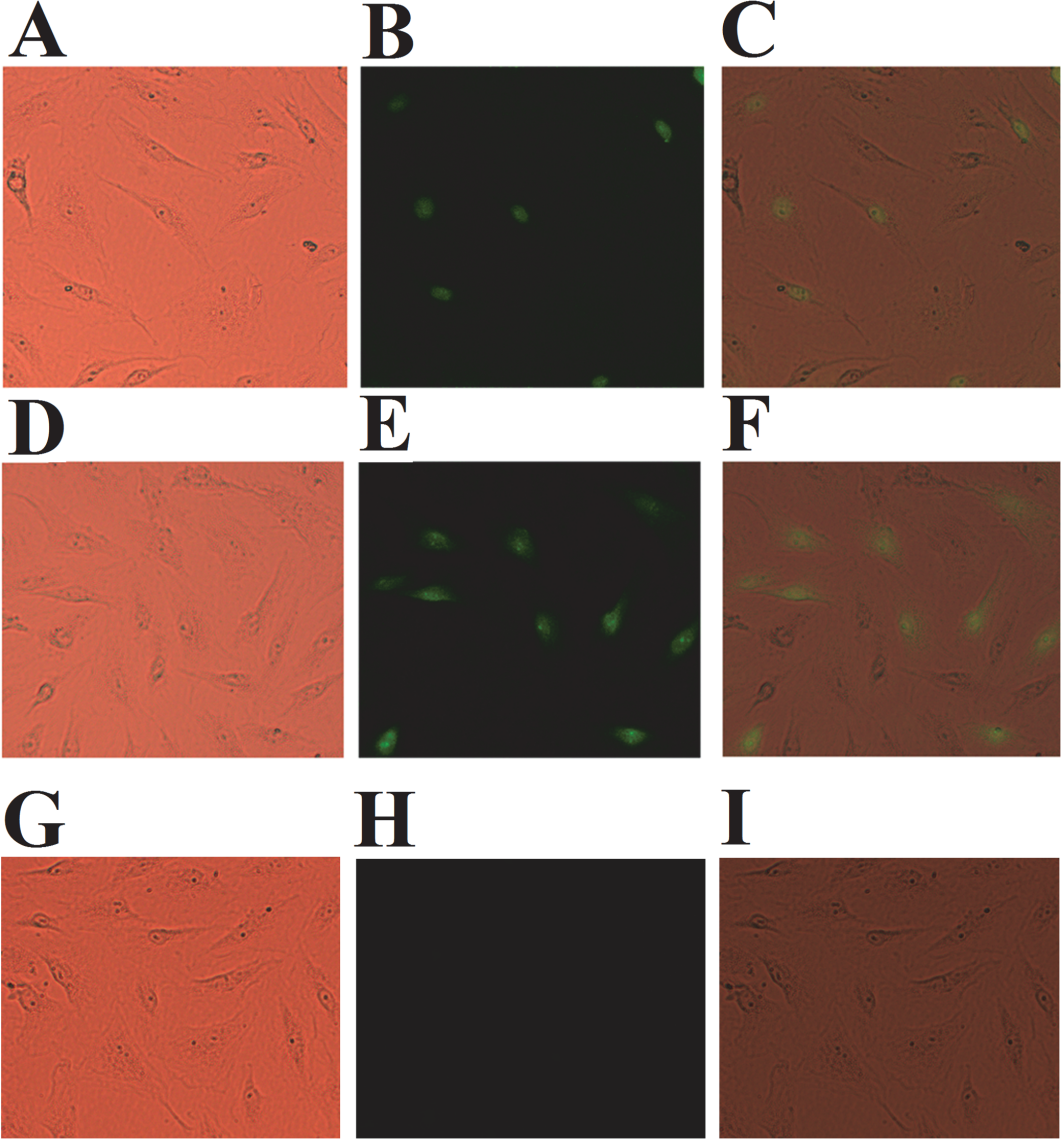
Indirect immunofluorescence analysis of the expression of His-tagged P247 and P523 in GF cells transfected with pCN247 and pCN523. pCN247 (A and B), pCN523 (D and E), and pCN3 (G and H) transfectants were incubated with mouse anti-His monoclonal antibody and then with FITC-labeled goat anti-mouse antibody. The cells were observed under a microscope with (B, E, and H) or without (A, D, and G) fluorescence. Panels C, F, and I are merges of A and B, D and E, and G and H respectively. Magnification, 25 × 5.

In a preliminary screening study, in order to identify megalocytivirus genes with immunoprotective potential, we constructed 22 DNA vaccine plasmids based on 22 different genes of megalocytivirus RBIV-C1. The protective effect of these vaccines was examined in a turbot model. The results showed that fish vaccinated with two of the vaccine plasmids, pCN247 and pCN523, exhibited high survival rates (over 60%). pCN247 and pCN523 were constructed based on the ORF107 and ORF86, respectively, of megalocytivirus RBIV-C1. ORF107 encodes a putative nuclear antigen of 247 amino acid residues (named P247), while ORF86 encodes a hypothetical protein of 523 amino acid residues (named P523) with no known function or conserved domain structure. The ability of pCN247 and pCN523 to express the vaccine genes in fish cells was verified by IFA assay, which showed that following transfection into GF cells (a fish cell line), pCN247 and pCN523, but not the control plasmid pCN3, were able to express the encoded vaccine proteins in the transfectants (Fig. 1). With these preliminary results, we then selected pCN247 and pCN523 for further study.

### Natural production of P247 and P523 in megalocytivirus-infected fish

**Fig 2:**
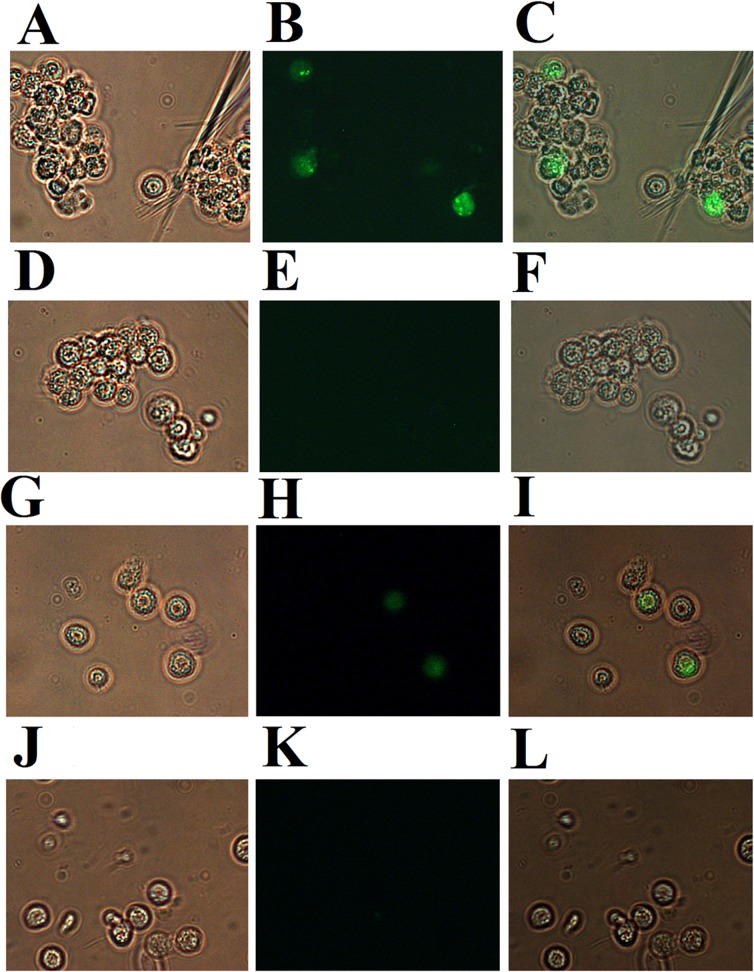
Immunofluorescent analysis of P247 and P523 expression in megalocytivirus-infected fish. Peripheral blood leukocytes were collected from turbot infected with (A, B, G, and H) or without (D, E, J, and K) megalocytivirus. The cells were treated with rat antibodies against recombinant P247 (A, B, D, and E) or P523 (G, H, J, and K) and then with FITC-labeled goat anti-rat antibodies. The cells were observed under a microscope with (B, E, H, and K) or without (A, D, G, and J) fluorescence. Panels C, F, I, and L are merges of A and B, D and E, G and H, and J and K respectively. Magnification, 10×40.

Before further study of P247 and P523, we examined whether these two proteins were actually expressed by megalocytivirus during infection. For this purpose, PBL from megalocytivirus-infected turbot were subjected to immunofluorescence analysis with antibodies against recombinant P247 and P523. The results showed that for both P247 and P523 detections, fluorescence was observed in the PBL from megalocytivirus-infected fish but not in the PBL from the uninfected control fish (Fig. 2), suggesting that P247 and P523 were indeed produced by the virus in the infected host.

### Vaccination of turbot with pCN247 and pCN523

**Fig 3:**
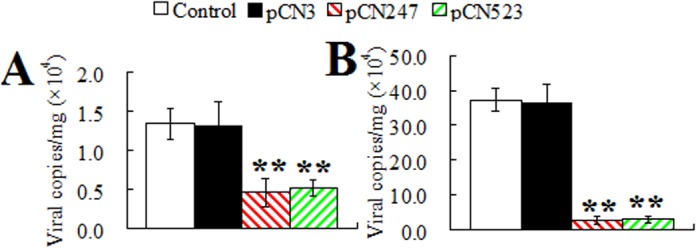
Viral replication in vaccinated fish. Turbot were vaccinated with pCN247, pCN523, pCN3, or PBS (control) and challenged with megalocytivirus at one month post-vaccination. Viral loads in spleen were determined at 3 days (A) and 5 days (B) post-challenge. Data are shown as means ± SE (N = 3). N, the number of times the experiment was performed. **P < 0.01.

**Fig 4:**
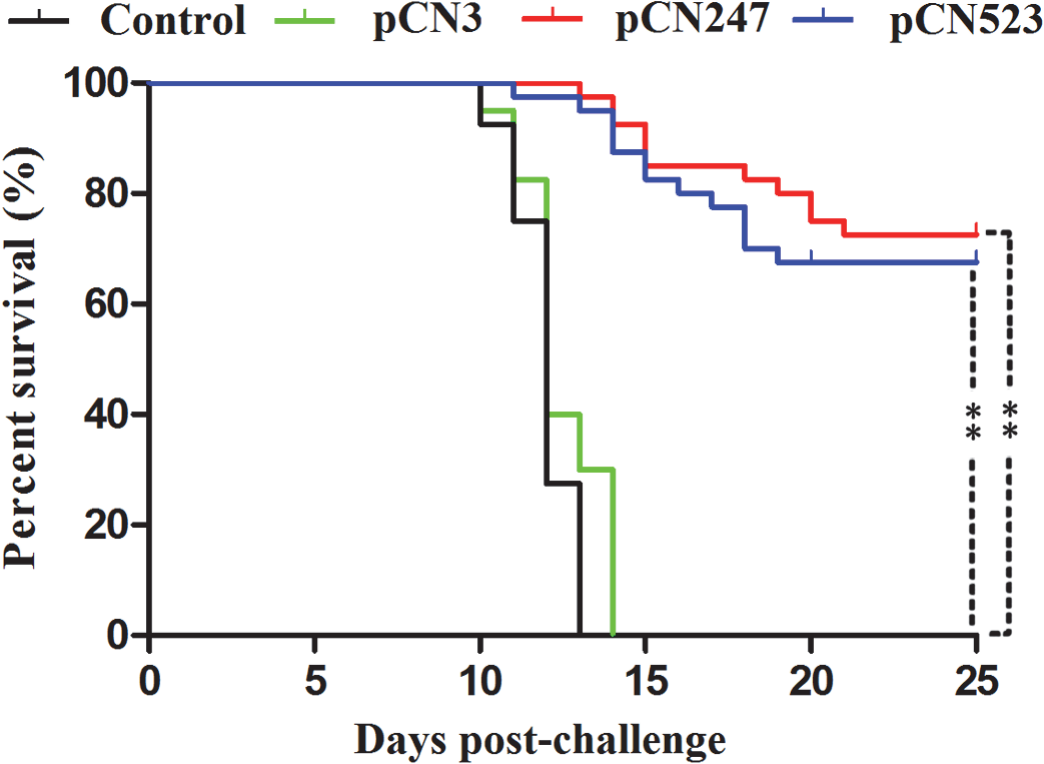
Survival of vaccinated fish. Turbot vaccinated with pCN3, pCN247, pCN523, and PBS (Control) were challenged with megalocytivirus and monitored daily for survival. Significance between the survivals of the vaccinated fish and the control fish was determined with logrank test. **P < 0.01.

- **Expression of the vaccine genes in fish tissues.** To confirm the immunoprotective potential of pCN247 and pCN523 observed in the preliminary selection described above, two repeat vaccination trials were conducted, in which turbot were immunized with pCN247, pCN523, the control vector pCN3, or PBS. Distribution of the DNA vaccine plasmids in fish tissues was determined by PCR at 7 days post-vaccination. The results showed that pCN247, pCN523, and pCN3 were detected in the kidney, spleen, and muscle of the fish vaccinated with the respective plasmids, whereas no plasmid was detected in PBS-vaccinated fish (S2 Fig. and data not shown). To examine whether the vaccine genes were expressed in fish tissues, RT-PCR was performed to determine the mRNA levels of P247 and P523 at 7 days post-vaccination. The results showed that P247 and P523 mRNA transcripts were detected in the muscle, kidney, and spleen of the fish vaccinated with pCN247 and pCN523 respectively, but not in fish vaccinated with pCN3 or PBS (S2 Fig. and data not shown). These results indicate that the vaccine genes were successfully expressed in the immunized fish.
- **Protection induced by pCN247 and pCN523.** To examine the protective efficacy of pCN247 and pCN523, the vaccinated fish were challenged with megalocytivirus at one month post-vaccination and monitored for mortality and for viral replication in spleen at 3 days and 5 days post-viral challenge. The results showed that, compared to fish vaccinated with pCN3 and PBS, fish vaccinated with pCN247 and pCN523 exhibited significantly reduced viral loads at both time points (Fig. 3). The survival rates of the fish vaccinated with pCN247, pCN523, pCN3, and PBS were 75% (30/40), 63% (25/40), 0%, and 0% respectively (Fig. 4). Based on these results, the protection rates, in terms of RPS, induced by pCN247 and pCN523 were 75% and 63% respectively with PBS as a control. Megalocytivirus RBIV-C1 was detected in the kidney, spleen, and liver of dying fish, which confirmed cause of death by viral infection. Comparable protection rates (70% and 65% for pCN247 and pCN523 respectively) were obtained from the repeat vaccination trial.

### Immune response induced by pCN247 and pCN523

**Fig 5:**
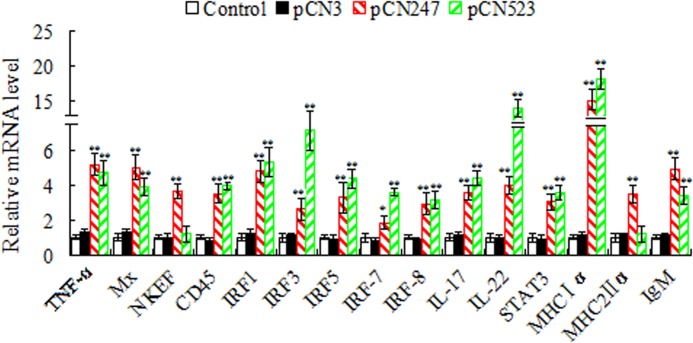
Immune gene expression in vaccinated fish. Turbot were vaccinated with or without (control) pCN247, pCN523, or pCN3 and challenged with megalocytivirus. At 24 h post-challenge, immune gene expression in spleen was determined by quantitative real time RT-PCR. For convenience of comparison, for each gene the mRNA level of the control fish was set as 1. Data are presented as means±SE (N = 3). N, the number of times the experiment was performed. **P < 0.01; *P < 0.05.

**Fig 6:**
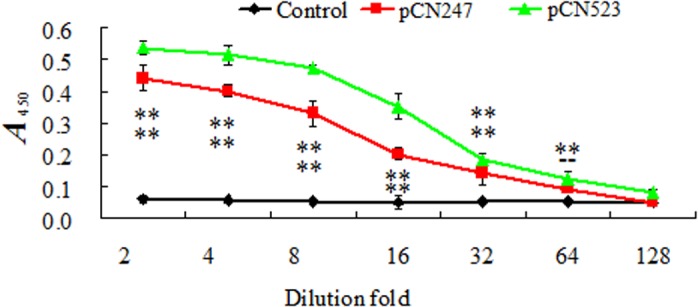
Serum antibody production in vaccinated fish. Sera were collected from turbot vaccinated with or without (control) pCN247 or pCN523. The sera were diluted in different folds, and serum antibodies against rP247 and rP523 were determined by ELISA. Data are presented as means±SE (N = 3). N, the number of times the assay was performed. At each dilution, significances between vaccinated fish and control fish are indicated by asterisk. **P < 0.01.

**Fig 7:**
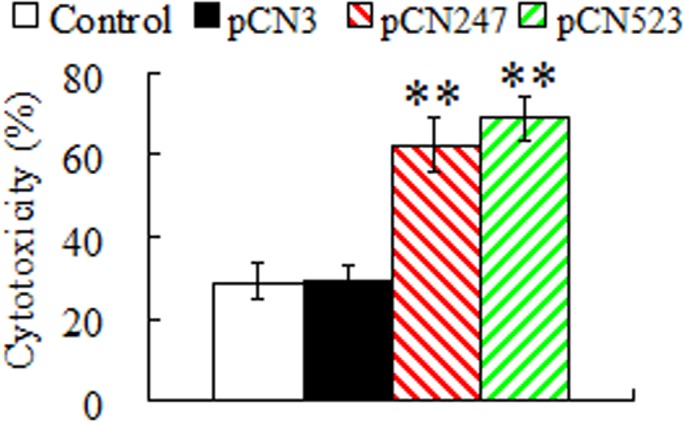
Cytotoxicity of peripheral blood leukocytes (PBL) of vaccinated fish. PBL from turbot vaccinated with or without (control) pCN247, pCN523, or pCN3 were used as effector cells, while PBL from megalocytivirus-infected turbot were used as target cells. The effector and target cells were mixed and incubated for 24 h. The cytotoxicity of the effector cells was determined by lactate dehydrogenase assay. Data are presented as means ± SE (N = 3). N, the number of times the experiment was performed. **P < 0.01.

**Fig 8:**
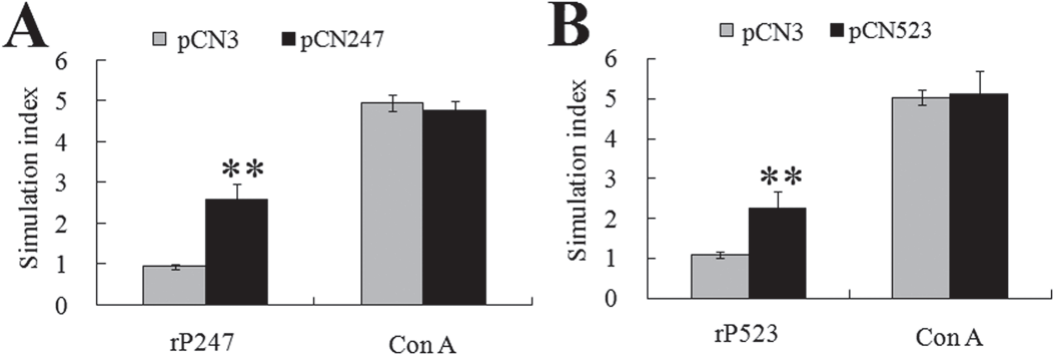
Proliferative activity of peripheral blood leukocytes (PBL) from vaccinated fish in response to antigen stimulation. PBL from turbot vaccinated with pCN247, pCN523, or pCN3 were treated with or without (control) rP247, rP523, or ConA, and cellular proliferation was determined by MTT assay. Stimulation index was defined as fold increase in the proliferation of antigen-treated cells compared to that of the untreated control cells. Data are presented as means ± SE (N = 3). N, the number of times the experiment was performed. **P < 0.01.

- **Immune gene expression.** At 24 h post-viral challenge, qRT-PCR was conducted to determine the expression of immune genes in spleen. The genes examined are tumor necrosis factor-α (TNF-α), myxovirus-resistant (Mx), nature killer enhancing factor (NKEF), leukocyte common antigen CD45, interferon regulatory factor (IRF) 1, IRF3, IRF5, IRF7, IRF8, interleukin (IL)-1β, IL-8, IL-17, IL-22, signal transducer and activator of transcription 3 (STAT3), major histocompatibility complex (MHC) Iα, MHCIIα, immunoglobulin (Ig) M, and IgD. The results showed that the expression levels of TNF-α, Mx, NKEF, CD45, IRF1, IRF3, IRF5, IRF7, IRF8, IL-17, IL-22, STAT3, MHCIα, MHCIIα, and IgM in pCN247-vaccinated fish were significantly upregulated (Fig. 5). The expression levels of IL-1β, IL-8, and IgD, however, were comparable to those in the pCN3- or PBS-vaccinated fish (data not shown). In pCN523-vaccinated fish, except for IL-1β, IL-8, IgD, NKEF, and MHCIIα, all other genes were upregulated to significant extents.
- **Production of specific serum antibodies.** To examine serum antibody production, sera were collected from the fish vaccinated with pCN247, pCN523, pCN3, or PBS at one month post-vaccination. The sera were diluted in different folds and subjected to ELISA analysis. The results showed that for the sera from pCN247-vaccinated fish, specific antibodies were detected in the 32-fold and lower fold dilutions, while for the sera from pCN523-vaccinated fish, specific antibodies were detected in the 64-fold and lower fold dilutions (Fig. 6). No specific serum antibodies were detected in pCN3-vaccinated fish.
- **Generation of cytotoxic immune cells in PBL.** To examine pCN247- and pCN523-induced cellular immune response, the cytotoxicity of the PBL from vaccinated fish was assessed. For this purpose, PBL were collected from turbot vaccinated with pCN247, pCN523, pCN3, or PBS. In subsequent LDH assay, these PBL served as effector cells and were incubated with the target PBL from megalocytivirus-infected turbot. The results showed that compared to the cytotoxic activities of the PBL from PBS- and pCN3-vaccinated fish, which were comparable, the cytotoxic activities of the PBL from pCN247- and pCN523-vaccinated fish were significantly higher (Fig. 7).
- **Responsiveness of PBL to secondary antigen stimulation.** To examine the effect of vaccination on the production of memory immune cells, PBL from pCN247-, pCN523-, pCN3-vaccinated fish were treated with rP247, rP523, or ConA, and the proliferative response of the cells was measured by MTT assay. The results showed that following rP247 treatment, the proliferative activity of the PBL from pCN247-vaccinated fish was significantly increased compared to that of the PBL from pCN3-vaccinated fish (Fig. 8A). In contrast, following ConA treatment, the proliferative activities of the PBL from both pCN247- and pCN3-vaccinated fish were increased to comparable levels. Similarly, rP523 treatment induced significantly higher proliferation in the PBL from pCN523-vaccinated fish than in the PBL from pCN3-vaccinated fish, whereas ConA treatment caused no apparent difference in the proliferation of the PBL from pCN523- and pCN3-vaccinated fish (Fig. 8B).

### Potential role of P247 and P523 in viral infection

**Fig 9:**
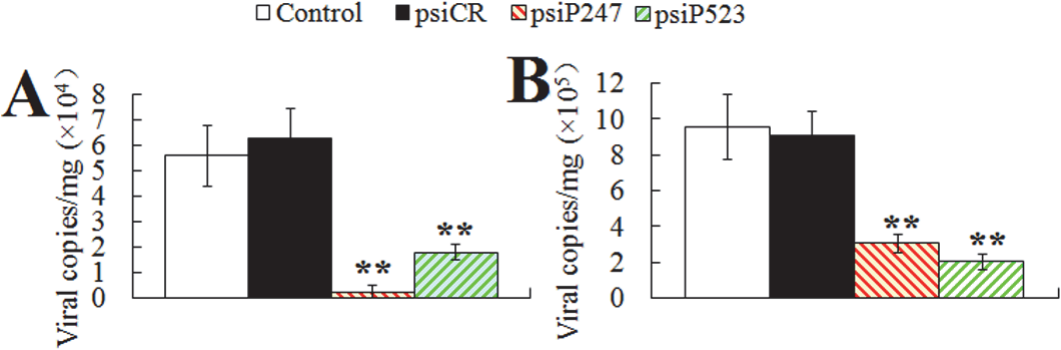
Effect of P247 and P523 knockdown on viral infection. Turbot administered with PBS (control), psiP247, psiP523, or psiCR were infected with megalocytivirus, and the amount of virus in spleen was determined at 3 days (A) and 5 days (B) after infection. Data are expressed as the mean ± SE (N = 3). N, the number of times the experiment was performed. **P < 0.01.

**Fig 10:**
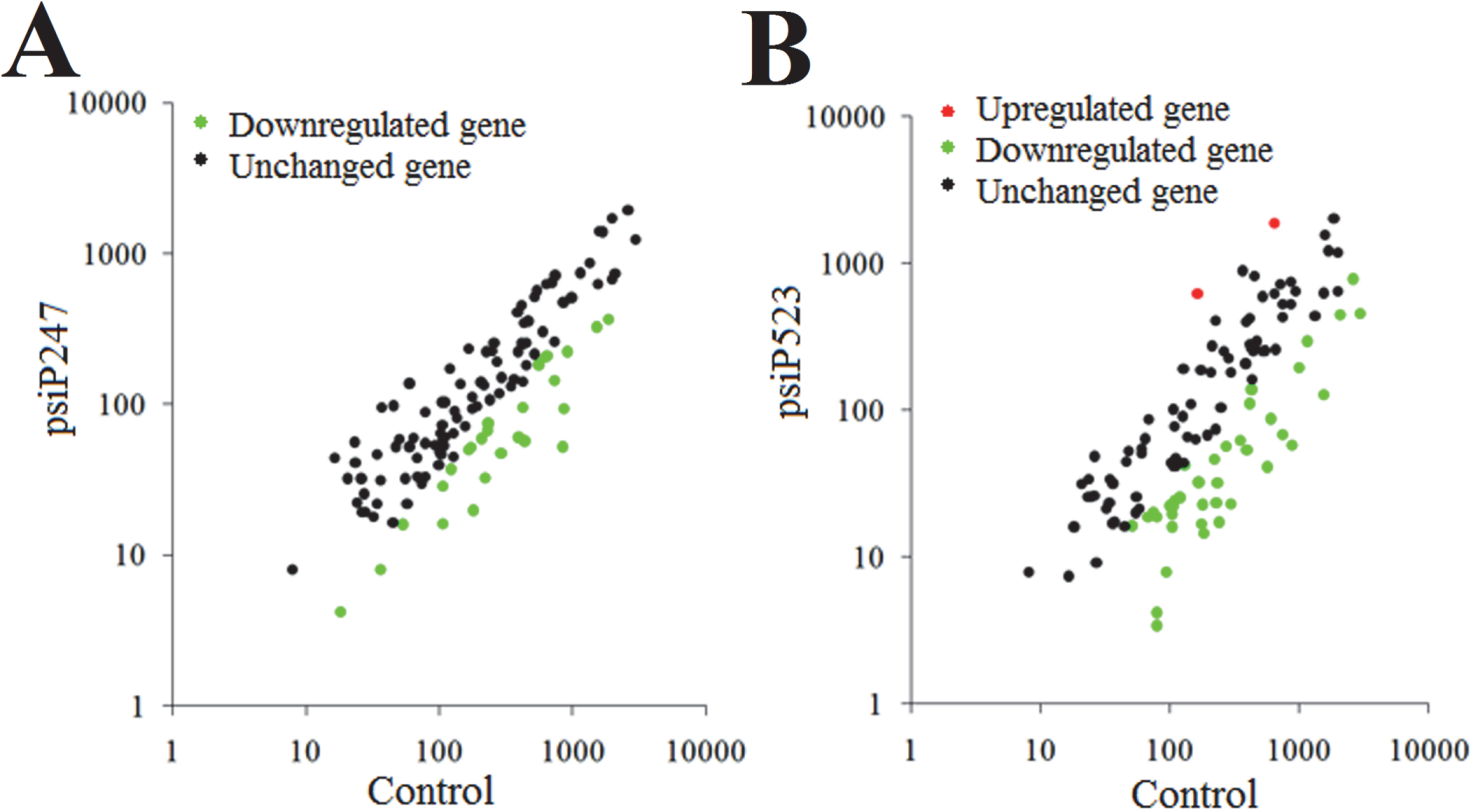
Effect of P247 and P523 knockdown on viral gene expression on a global scale. Turbot administered with psiP247 (A), psiP523 (B), or psiCR (control) were infected with megalocytivirus, and the expression of 119 viral genes in the spleen was determined by quantitative real time RT-PCR.

- **P247 and P523 knockdown.** So far our results indicate that P247 and P523, in the form of DNA vaccine, induced protective immunity against megalocytivirus. Based on these results, we hypothesized that P247 and P523, whose functionalities as viral components were entirely unknown, may possibly be essential factors in viral replication. To investigate this hypothesis, we examined the effect of P247 and P523 knockdown on megalocytivirus infection. For this purpose, the plasmids psiP247 and psiP523 were constructed, which were designed to express *in vivo* P247- and P523-specific siRNA respectively. As a control, the plasmid psiCR was also created, which expresses a nonspecific siRNA. To examine the interfering efficiency of the siRNA, turbot administered with or without psiP247, psiP523, or psiCR were infected with megalocytivirus, and the expression of P247 and P523 was determined by qRT-PCR at 5 days post-infection. The results showed that the expression of P247 in psi247-admisnitered fish was significantly (*P* < 0.01) reduced to the level of 54% of that in the control fish. Likewise, the expression level of P523 in psi523-admisnitered fish was significantly (*P* < 0.01) reduced to 41% of that in the control fish. In contrast, the expression levels of P247 and P523 in psiCR-administered fish were comparable to those in the control fish. These results indicate that psiP247 and psiP523 effectively reduced the expressions of P247 and P523 respectively.
- **Effect of P247 and P523 knockdown on viral replication.** To examine the effect of P247 and P523 knockdown on viral replication, turbot administered with psiP247, psiP523, psiCR, or PBS were infected with megalocytivirus, and the viral load in spleen was determined at 3 days and 5 days post-infection. The results showed that the presence of psiP247 and psiP523 significantly reduced viral numbers at both time points, whereas the presence of psiCR had no apparent effect on viral replication (Fig. 9).
- **Effect of P247 and P523 knockdown on viral gene expression on a global scale.** With the above result, which showed that interference with P247 and P523 expression affected viral replication, we wondered whether P247 and P523 disregulation would have any impact on the expression of viral genes on a global scale. To investigate this question, we conducted a whole-genome transcriptome analysis to examine the effect of P247- and P523-knockdown on the expression of all putative genes of megalocytivirus RBIV-C1 as represented by the 119 ORFs identified in the genome of the virus. The results showed that compared to control fish, psiP247-administered fish exhibited significant (*P* < 0.01) changes (2.8- to 16-fold) in the expression of 26 genes, all which being downregulated (Fig. 10; Table 2). Of these genes, three (ORF 18L, 34L, and 51L) are putative envelop proteins, one (ORF 74L) is protein kinases/phosphatases, three (ORF 16R, 113L, and 116L) are regulatory proteins, three (ORF 21R, 47L, and 83R) are associated with DNA replication, two (ORF 63L and 72R) are involved in RNA transcription. The remaining 14 down-regulated genes (ORF 17L, 22L, 28L, 41L, 46L, 52L, 65L, 66R, 77L, 78R, 82L, 90L, 100R, and 102L) are unknown in function. In psiP523-administered fish, significant (*P* < 0.01) changes (2.6- to 44-fold) in the expression of 41 genes were observed, including 39 down-regulated genes and 2 up-regulated genes (Fig. 10; Table 2). Of the down-regulated genes, two (ORF 18L, and 19R) are putative envelop proteins, four (ORF 6L, 24L, 55L, and 74L) are protein kinases/phosphatases, three (ORF 16R, 98R, and 106L) are regulatory proteins, one (ORF 13R) is ubiquitin ligases, two (ORF 44L and 105R) are functional enzymes, three (ORF 21R, 29L, and 47L) are associated with DNA replication, one (ORF 35R) is associated with RNA transcription, and three (ORF 2L, 25R, and 59L) are involved in cell proliferation. The remaining 20 down-regulated genes (ORF 9R, 11L, 15R, 17L, 23L, 27R, 38L, 39L, 42L, 46L, 48R, 52L, 53R, 54L, 57L, 58L, 72R, 81R, 82L, and 103L) are unknown in function. And the two (ORF 75R and 113L) up-regulated genes are associated with cell regulation activity.

**Table 2:** Genes affected in expression by P247 and P523 knockdown.

P247 knockdown		P523 knockdown		Putative function
Up^a^	Down^b^	Up	Down	
	ORF18L, ORF34L, ORF51L		ORF18L, ORF19R,	Envelop protein
	ORF74L		ORF6L, ORF24L, ORF55L, ORF74L	Kinase/Phosphatase
	ORF16R, ORF113L, ORF116L,	ORF75R, ORF113L	ORF16R, ORF98R, ORF106L	Regulatory protein
	ORF21R, ORF47L, ORF83R		ORF21R, ORF29L, ORF47L	DNA replication protein
	ORF63L, ORF72R,		ORF35R	RNA transcription Protein
			ORF2L, ORF25R, ORF59L	Cell proliferation Protein
			ORF13R	Ubiquitin ligase
			ORF44L, ORF105R	Other enzyme
	ORF17L, ORF22L, ORF28L, ORF41L, ORF46L, ORF52L, ORF65L, ORF66R, ORF77L, ORF78R, ORF82L, ORF90L, ORF100R, ORF102L		ORF9R, ORF11L, ORF15R, ORF17L, ORF23L, ORF27R, ORF38L, ORF39L, ORF42L, ORF46L, ORF48R, ORF52L, ORF53R, ORF54L, ORF57L, ORF58L, ORF72R, ORF81R, ORF82L, ORF103L	Unknown protein

^a^Up: regulated

^b^Down: downregulated.

## Discussion

In this study, we examined the vaccine potentials of a set of megalocytivirus genes as DNA vaccines based on the knowledge that DNA vaccine has the unique feature of stimulating both humoral and cellular immune responses, which are important in the control of viral diseases [39–41]. Of the 22 viral genes examined, two genes expressed from the vaccine plasmids pCN247 and pCN523 induced effective protection. For DNA vaccines, one fundamental requirement is that the vaccine genes have to be expressed in the target host. In our study, immunofluorescence microscopy showed that fish cells transfected with pCN247 and pCN523 were able to produce recombinant P247 and P523 respectively, suggesting that the exogenous vaccine genes were expressed in the transfectants. Consistent with this observation, pCN247 and pCN523 were localized in multiple tissues following vaccination into turbot, and P247 and P523 expression in tissues was detected by qRT-PCR. These results are similar to those observed in previous studies of fish DNA vaccines [23,28,42–44], which indicate that DNA vaccines dictated by a mammalian promoter can be successfully expressed in teleost via the host transcription and translation system. Protection analysis showed that following challenge with megalocytivirus, turbot vaccinated with pCN247 and pCN523 exhibited significantly reduced viral loads, suggesting that pCN247 and pCN523 must have induced certain immune responses that inhibited viral replication. In line with these observations, pCN247 and pCN523 conferred protection rates of over 63%, which are striking, given the fact that the accumulated mortality of the control fish was 100%. These results indicate that pCN247 and pCN523 are highly protective vaccine candidates. Although the protection test was conducted with turbot, the results may be applied to other teleost species. Similar to most fish vaccine studies, which usually involve much larger amounts of animals than that in kindred studies with mammals, the number of animals used in our vaccination trial was relatively large. In future studies, the amount of experimental fish may be reduced to minimize the killing of animals.

In mammals, cell-mediated cytotoxicity, in which virus-infected cells are recognized and lysed by effector cells of the immune system, is a vital mechanism in combating viral infections [45,46]. In fish, CD8(+) cytotoxic T lymphocytes (CTLs) similar to those of mammalian systems have been identified, and there are evidences that indicate the existence of cell-mediated immunity [47–49]. It has been reported that PBL isolated from rainbow trout vaccinated with a DNA vaccine coding for the G protein of VHSV exhibited increased number of CTLs, and that PBL from rainbow trout sub-lethally infected with VHSV killed MHC class I-matched as well as xenogeneic MHC class I-mismatched VHSV-infected cells [50,51]. In studies with red sea bream, cell-mediated cytotoxicity was important for preventing red sea bream iridovirus (RSIV) infection [29]. In our study, we found that PBL from pCN247- and pCN523-vaccinated fish possessed significantly enhanced cytotoxic activity against cells from megalocytivirus-infected fish, suggesting that vaccination with pCN247 and pCN523 elicited cell-mediated immunity that generated megalocytivirus-targeting CTLs. In line with this observation, proliferation analysis showed that compared to PBL from the control fish, PBL from pCN247- and pCN523-vaccinated fish exhibited similar levels of response to ConA stimulation but significantly stronger response to rP247 and rP523 stimulation. These results suggest that vaccination induces the production of P247- and P523-specific memory cells in PBL that recognize and are activated by the specific antigens upon secondary encounter.

Previous studies have shown that fish DNA vaccines promote the expression of immune genes in a manner that depends on the vaccine and the target animal [24,52–55]. In this study, qRT-PCR showed that in both pCN247- and pCN523-vaccinated fish, genes of innate and adapted immunity were significantly upregulated in expression, including those known to be involved in antiviral response (Mx, NKEF, and IRF series), which probably accounts in part for the reduced viral burdens observed in the vaccinated fish. Consistent with the significantly upregulated expression of IgM in pCN247- and pCN523-vaccinated fish, specific serum antibodies were detected in these fish. These results indicate that pCN247 and pCN523 elicited both humoral and cellular immune responses in turbot.

Accumulating evidences have shown that siRNA can specifically and potently inhibit a number of viruses, including human immunodeficiency virus [56], West Nile virus [57], Japanese encephalitis virus [58], and influenza virus [59]. Likewise, in the present study, we found that the siRNA expressed from psiP247 and psiP523 effectively reduced the expression of P247 and P523, respectively, to levels comparable to those reported previously in other teleost species [60–63]. Unlike the observations made with mammals, in which siRNA can induce very high levels of inhibition on the expression of target genes, the efficiencies of siRNA in teleost are generally around 40%-60% [60,61,64]. Nevertheless, significant effects can be ensued from the relatively moderate RNA interference [60–64]. In our study, *in vivo* infection showed that P247 and P523 knockdown significantly impaired viral replication in fish tissues, suggesting that the normal expression of P247 and P523 are essential to viral infection. These results are not in conflict with those of pCN247 and pCN523 vaccination. In the latter case, vaccination of pCN247 and pCN523 led to production of P247 and P523 in fish at least by 7 days post-vaccination, which enabled induction of host immune response, such as generation of CTLs and antibodies specific to P247 and P523 as said above. When the fish were challenged with the virus at one month post-vaccination, the P247 and P523 expressed by the virus were recognized and blocked in function by host immune factors (e.g. antibodies that neutralize P247 and P523), and virus-infected host cells expressing P247 and P523 were killed by specific CTLs. As a result, overall viral replication was reduced. In line with these observations, whole-genome transcription analysis showed that P247 and P523 knockdown altered the expression of a large number of viral genes. It is noteworthy that all 26 genes affected by P247 knockdown were downregulated; likewise, the majority of P523-modulated genes were also downregulated. These results indicate that under normal conditions, P247 and P523 are required for the optimal expression of these “target” genes, which suggests a direct or indirect stimulatory role of P247 and P523 as far as the expression of these genes is concerned. Since the affected genes cover the essential aspects of viral replication (envelop protein synthesis, DNA replication, RNA transcription etc), their attenuated expression inevitably impairs viral infection.

In conclusion, in this study we identified two megalocytivirus proteins, P247 and P523, which in the form of DNA vaccines elicit effective protective immunity. Our results indicate production of cytotoxic and memory immune cells and thus an involvement of specific cellular defense in P247- and P523-vaccianted fish, which add insights to the immune mechanism of DNA vaccines in teleost. In addition, we demonstrate that P247 and P523 play fundamental roles essential to viral replication, which promotes our understanding of the infection of megalocytivirus and suggests for the first time a link between the protectivity of viral immunogens and their biological significance in viral replication.

## Supporting Information

S1 Fig SDS-PAGE of purified recombinant P247 (corresponding to positions 47–217) (lane 2) and P523 (corresponding to positions 115–305) (lane 3). Purified proteins were analyzed by SDS-PAGE and viewed after staining with Coomassie brilliant blue R-250. Lane 1, protein markers.
(TIF)

S2 Fig Presence of vaccine plasmids (A) and expression of the vaccine-encoding genes (B) in fish tissue. (A) Turbot were vaccinated with pCN3 (lane 2), pCN523 (lane 4), pCN247 (lane 6), and PBS (lanes 3, 5, and 7). At 7 days post-vaccination, DNA was extracted from spleen and used for PCR with primers specific to pCN3 (lanes 2 and 3), pCN523 (lanes 4 and 5), and pCN247 (lanes 6 and 7). (B) Turbot were vaccinated with pCN523 (lane 2), pCN247 (lane 5), pCN3 (lanes 3 and 6), and PBS (lanes 4 and 7). At 7 days post-vaccination, RNA was extracted from spleen and used for RT-PCR with primers specific to plasmid-derived P523 (lanes 2, 3, and 4 of the upper panel), P247 (lanes 5, 6, and 7 of the upper panel), or, as an internal control, with primers specific to RNA polynerase II subunit D (RPSD) (lower panel). Lane 1 of both panels, DNA molecular weight markers.
(TIF)
