# Supplementary figures and images for: P247 and P523: Two In Vivo-Expressed Megalocytivirus Proteins That Induce Protective Immunity and Are Essential to Viral Infection
Source: PLoS One. 2015 Mar 27;10(3):e0121282. doi: 10.1371/journal.pone.0121282 (PMC4376877; doi:10.1371/journal.pone.0121282)

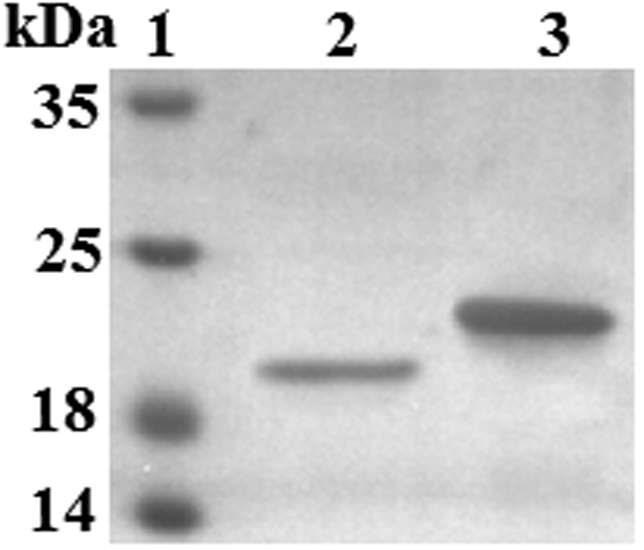

Supplement: S1 Fig — Purified proteins were analyzed by SDS-PAGE and viewed after staining with Coomassie brilliant blue R-250. Lane 1, protein markers. (TIF) [file pone.0121282.s001.tif]

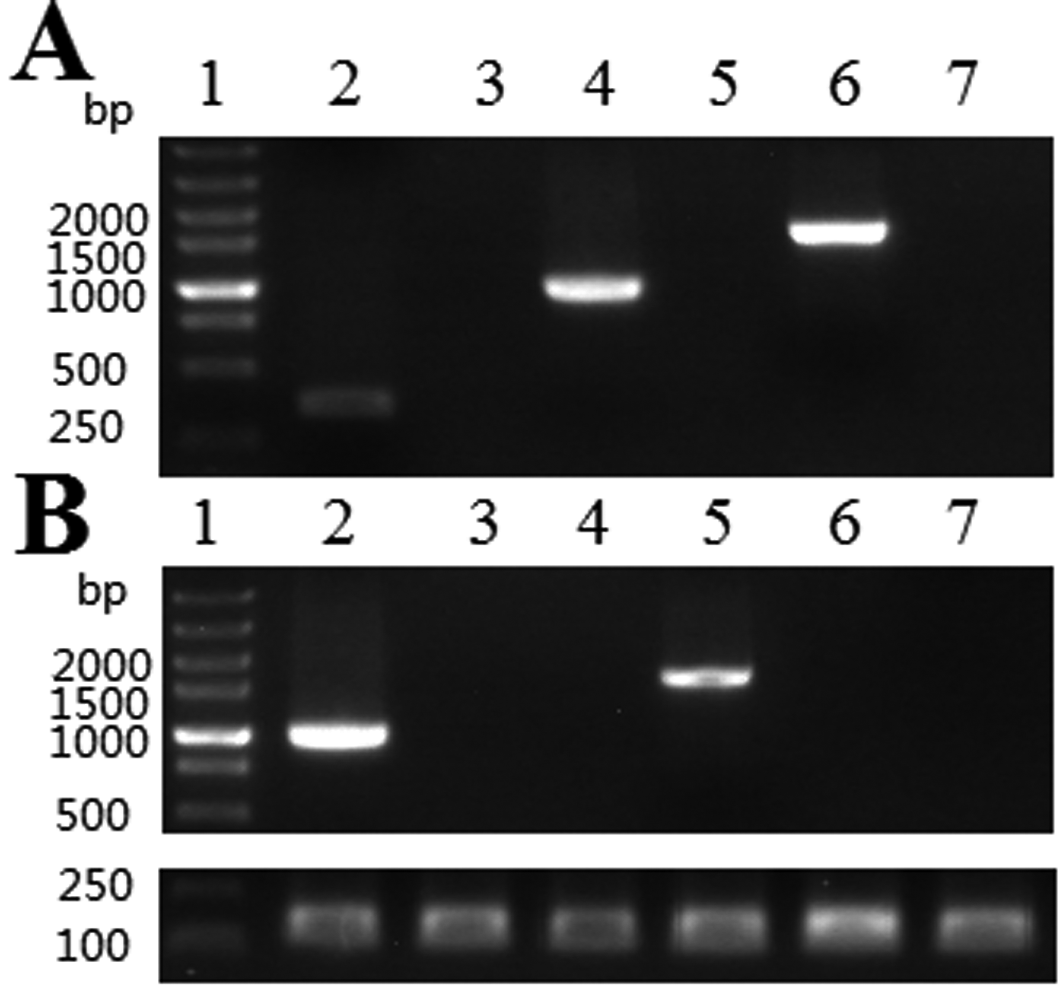

Supplement: S2 Fig — (A) Turbot were vaccinated with pCN3 (lane 2), pCN523 (lane 4), pCN247 (lane 6), and PBS (lanes 3, 5, and 7). At 7 days post-vaccination, DNA was extracted from spleen and used for PCR with primers specific to pCN3 (lanes 2 and 3), pCN523 (lanes 4 and 5), and pCN247 (lanes 6 and 7). (B) Turbot were vaccinated with pCN523 (lane 2), pCN247 (lane 5), pCN3 (lanes 3 and 6), and PBS (lanes 4 and 7). At 7 days post-vaccination, RNA was extracted from spleen and used for RT-PCR with primers specific to plasmid-derived P523 (lanes 2, 3, and 4 of the upper panel), P247 (lanes 5, 6, and 7 of the upper panel), or, as an internal control, with primers specific to RNA polynerase II subunit D (RPSD) (lower panel). Lane 1 of both panels, DNA molecular weight markers. (TIF) [file pone.0121282.s002.tif]
